# Engaging Caregivers, Older Adults and Community Members in Oral Health Education: The MOTIVATE at Home Pilot Program

**DOI:** 10.1093/geroni/igaf122.4379

**Published:** 2025-12-31

**Authors:** Jennifer Crittenden, Denise O’Connell, Tara Wilson, Labrini Nelligan

**Affiliations:** University of Maine Center on Aging, Orono, Maine, United States; Massachusetts General Hospital, Bangor, Maine, United States; Massachusetts General Hospital, Bangor, Maine, United States; Massachusetts General Hospital, Bangor, Maine, United States

## Abstract

The MOTIVATE at Home (MAH) program brings education to caregivers so they are empowered to provide and/or support oral health care for older adults living at home, a growing population that disproportionately faces oral health disparities and health risks. The MAH pilot included statewide online and in-person educational sessions, learning stations at community-based organizations, and online “Ask the Dental Expert” sessions. Surveys were collected from 121 learners across MAH offerings with six additional qualitative interviews conducted with partnering community-based host sites. The audience ranged in age from 23-94 years old (M = 68 years) with 28% reporting a current caregiving role. The majority of participants (86%) learned something new that they could use to improve their own oral health and 83% of all participants, regardless of current self-reported care partner status, reported learning something new that they could use to improve the oral health of someone for who they provide care. Key findings demonstrate the value of a community-based accessible approach to education. Partners also reported that the MAH program was a valuable addition to their current offerings and collaborations. Findings also underscore the importance of using a brief and engaging format for online learning, integration of staff roles and support into in-person learning stations to encourage participation, and inclusion of “gamification” and “fun” to enhance learning. Participants identified additional ongoing education needs focused on specialty dental topics, the connection between health/mental health and oral health, along with an ongoing need for access to affordable dental care for older adults.

